# The Progress of Cancer Research

**Published:** 1903-10-03

**Authors:** D'Arcy Power

**Affiliations:** Senior Surgeon to the Victoria Hospital for Children, Chelsea; Assistant Surgeon at St. Bartholomew's Hospital, E.C.


### The Progress of Cancer Research.

By D'ARCY POWER, F.R C.S.Eng., Senior Surgeon to the Victoria Hospital for Children, Chelsea; Assistant Surgeon at St. Bartholomew's Hospital, E.C.

Both at home and abroad much good work has been done within the last few years to discover the cause of cancer. At home the interest which the King is known to take in the subject has turned popular attention to the question in a manner which is apt to be embarrassing to those engaged upon original work. People who know little of the disease in any of its protean aspects and less of the difficulties attending any investigation carried out in a scientific manner write freely and publicly about cancer with a display of appalling ignorance which would be amusing if it did not encourage false hopes in the victims of cancer. Hopes that too often lead them to postpone a surgical operation which in the present state of our knowledge is the only way in which relief can be offered. Tentative results thus attain to a wholly unwarranted notoriety and a new cure is advertised daily. But this is only the embroidery of a fabric which is being carefully woven out of materials collected from a variety of sources, a fabric which, as it emerges from the loom, we hope will present for a pattern the cause as well as the cure of a very disastrous and widely-spread disease.

### The Pre-Scientific Era.

The marvel to those acquainted with the history of medicine is not so much our present ignorance of the cause of cancer as the amount of knowledge which has been collected within the last few years. The introduction of the microscope into medicine about 1850 first led to a clear differentiation of tumours, Virchow, Rokitanski, and Paget being the most notable workers. Before their time it was sufficient to know that a tumour was innocent or malignant, for it was impossible to refer the malignant growths to their proper positions, and it was often a mystery why apparently similar growths at one time ran a malignant course and at another time proved to be innocent. Gradually, however, the distinction of malignant growths into the two great groups of sarcoma, or connective tissue tumours, and carcinoma, or epithelial cancer, came to be clearly recognised, and the older clinical terms of recurrent fibroma and fungus hæmatodes disappeared from use. Very few surgeons, however, had received any scientific training, fewer still had been taught to think. The era of brilliant surgery had not yet passed away, for it belonged to the pre-anæsthetic days when a rapid operation was held in higher estimation than a radical operation, and a surgeon was still content to remove a malignant growth without staying to think whether by so doing he was removing the whole disease. Indeed, so muddily did he think that he brought himself to believe that recurrence was an inherent part of malignant disease, and he failed to understand that the subsequent death of the patient was due to the fact that his operation had failed because it was not sufficiently extensive. Even in scirrhus of the breast and in epithelioma of the tongue there were many surgeons as late as 1885 who were unable to appreciate the due relation of the cancer to the lymphatic glands. If the glands were so much enlarged that they could be felt, the axilla would be opened or the submental tissues would be incised, but the exploration of the lymphatic glands nearest to the growth was by no means a routine proceeding, and we owe to Sir Mitchell Banks the credit of enforcing the necessity for removing the whole breast, and with it the axillary glands in every case of mammary cancer, a method of proceeding which has been further extended by Professor Halsted.

### The Scientific Era.

For a long time the unaided work of individuals was alone directed to the investigation of cancer, and at first the outcome of thought about malignant disease was speculative rather than experimental. Pathologists and surgeons alike had forgotten John Hunter's aphorism, "Don't think, try." But out of these discussions as to the nature of tumours came the generalised conception of cancer, and later of malignant disease. The older observers had contented themselves with a careful description of malignant disease as it affected individual organs or structures, and did not realise, or at any rate did not discuss, malignant disease as an entity. This idea of the biological aspect of cancer I am inclined to attribute to Billroth and Cohnheim. But even their thoughts appear only to have extended to the human aspect, for the comparative study of disease was as yet in its infancy, and the backward state of the veterinary profession rendered it impossible to gain much information about malignant disease in animals.

The advent of bacteriology with the discovery that many diseases were associated with the presence of specific micro-organisms naturally raised a hope that cancer might be thus associated. Many investigations were carried out with this end in view, and with these investigations began the first serious attempts to ascertain the cause of cancer from a purely scientific standpoint. The early work was carried out on lines which had been successful with anthrax and tubercle and ended fruitlessly. It was left to Sir James Paget to suggest that if a micro-organism of cancer existed it might rather be of an animal than of a vegetable type—a protozoon and not a protophyte. Acting upon this hint, a number of observers immediately prepared and stained sections of cancerous tissues which showed to their own satisfaction and sometimes also to that of a few trusty adherents, that all sorts of parasites were present in all kinds of tissues. But none of the appearances thus demonstrated have fulfilled the hopes of their discoverers for they have not proved themselves capable either of producing the disease or of being cultivated outside the body. Indirectly the impetus given to the minute examination of carefully prepared animal tissues under high microscopic powers has been of great service, for it has added much to our knowledge of the life history of cells and somewhat to the diseases to which they are subject. We need not greatly regret, therefore, that ten years of the lives of many enthusiastic histologists were devoted to making themselves better acquainted with the minute structure of animal cells. The regret should rather be that in a manner wholly unworthy of science each proclaimed that he had found the parasite of cancer when in reality he had only succeeded in demonstrating certain unusual cellular appearances.

### The Infective Theory of Cancer.

The attempt to demonstrate the micro-organism of cancer naturally led to further investigation of this hypothesis on other than histological lines. Dr. Hanau, Messrs. Ballance and Shattock, Dr. Lack and myself have at various times and with varying success endeavoured to implant carcinomatous growths on healthy animals. Dr. Hanau and Dr. Lack each in a single instance seemed to have been successful, but Dr. Jensen alone has obtained thoroughly satisfactory results from this method of research. The work he records is in connection with cancer of the mouse. The original growth from which transplantations were made was a small tumour, whose structure was proved to be carcinomatous, growing on the back of a white mouse. Pieces of this growth were broken up in a mortar with sterile physiological salt solution, and a small amount of the fluid was injected subcutaneously into five mice. About 14 days after the injection a small nodule appeared, and increased in size with varying rapidity, in some cases developing to the size of half a hen's egg in the course of a few months, but there were no metastatic deposits. Further transplantations were made from some of the mice which had been thus inoculated, and up to the end of December, 1902, 844 mice had been so treated. Of these 232 died within 14 days of the inoculation. Of the remainder 274 were injected with material broken up in a mortar with salt solution, and in 121 tumours developed. In 338 mice a small piece of growth was inserted beneath the skin and in 128 of the animals growths developed. Thus about half of the injected mice gave positive results and 19 generations were inoculated. Dr. Jensen says that in the first few days after inoculation the material dwindled in size, but in about a fortnight fresh nodules appeared and gradually grew until in some cases the new growth was greater in weight than the rest of the animal. The condition of the injected material, too, influenced the result, the effect being greater when it was derived from the younger and smaller growths. It was further shown that all the animals were not equally susceptible and that individuals which resisted a first injection proved equally refractory to further inoculation; this seeming immunity was in some cases shown by whole families of mice. Dr. Jensen further studied the vitality of the tumour cells under various conditions. He found that they retained their vitality for only 24 hours if they were kept at the body temperature; summer heat, and the low temperature of 34–37° F. increased their resistance. An exposure of five minutes to a temperature of 115° F. and a few minutes to —4° F. killed the cells. Intense light also killed them, but only acted superficially. Partial drying was also destructive, and a quarter per cent. carbolic acid destroyed their vitality in five minutes. If these results, which are summarised from an abstract in a recent number of the *British Medical Journal* prove to be correct, Jensen's investigations will have carried us a long way on the road towards the discovery of the cause of cancer. But they are so pertinent and fit in so accurately with what we should expect to occur that they must be subjected to a further scrutiny before they can be accepted in their entirety.

This method of investigation needs further research before it can be said that the results were mere coincidences, for it is clear that cancer needs a peculiar soil upon which to flourish, and it is possible that the successful experiments were made with animals which happened to be predisposed to the disease. My own experiments were designed to ascertain whether irritation alone was a predisposing cause of cancer. For this purpose I kept the mucous membranes of various animals in a state of chronic irritation for many weeks, and I then brought them into contact with various malignant growths which had been freshly excised. I was careful to select very old and very young animals, and I endeavoured to inoculate them with cancer both from the human being and from other animals. But the results were always negative. In other experiments I saturated a given weight of earth with quantities of finely minced carcinomatous tissues which had been brought straight from the operating theatre, and I then kept a number of earthworms in the earth for many months, hoping by this means to discover if they would act as intermediate hosts for the suspected parasite of cancer; but the earthworms flourished, and I could never discover any abnormal appearances in their tissues. In like manner I have submitted flies captured in rooms where patients had been very ill of cancer to a very rigorous microscopical examination in the hope that their tissues might perhaps give some clue to the origin of cancer, because they are well-known transmitters of more than one disease, but in this case, too, the results of my examinations were purely negative, and I only found appearances which were capable of explanation on other than parasitic grounds.

The infective hypothesis of the cause of cancer is valuable as a stimulus to original work, but it has rather drawn away the thoughts from other fields which may prove even more fruitful, for as yet, except in Jensen's experiments, no results of any permanent value appear to have been obtained. The hypothesis has the disadvantage that infective, used in the scientific sense, is too often confounded by the general reader with infectious, used in the ordinary sense of the term. It has thus come about that cancer is said to be contagious, though there are no grounds for supposing that this is really so. Indeed, all evidence goes to prove the contrary. The nurses and attendants in hospital wards, devoted exclusively to the use of patients suffering from cancer, are not known to acquire the disease, and it is certain that surgeons who are constantly operating upon malignant disease are not more liable to suffer from cancer than those who are brought into less intimate contact with it.

### The Distribution of Cancer.

And yet there are many curious facts connected with cancer, all tending to show that if cancer is not contagious it is yet closely connected with locality, and perhaps with individuals. The geographical distribution of cancer in the broadest sense of the term is still unknown. It has been said that the natives of India, Jews, and men following such special occupations as that of a collier, are exempt from cancer. But the closer the inquiry the more certain it is that no such special exemption exists, and that the statements have been made either in ignorance or upon the observation of too small a number of cases. Our knowledge of the diseases which kill the savage races is not sufficiently complete to enable any general statement to be made as to the prevalence of malignant disease amongst them.

The local distribution of cancer in England and Wales occupied the attention of Mr. Alfred Haviland, who maintained in his book on "The Geographical Distribution of Disease in Great Britain" that cancer was unduly prevalent in those parts of the country where the soil was liable to become water logged. He mapped out the districts in England and Wales in which the cancer mortality was highest, and he showed that they coincided to a large extent with the river valleys, especially where there were clay soils and seasonal floods. He maintained on the other hand that cancer was infrequent in places characterised by elevated sites and limestone formations, or even in sites subject to floods, but within the immediate influence of calcareous rocks, because in these districts the soil does not become saturated with water. Mr. Haviland's generalisations were based almost entirely upon statistical evidence and they were soon found to be somewhat too absolute, though there is no doubt that they contain a considerable substratum of truth. Thus it was shown that cancer districts sometimes occur at comparatively high altitudes and that the two banks of the same river may show different aptitudes for its occurrence, whilst Mr. T. W. Blake called attention in 1895 to an interesting series of cases of malignant disease which occurred in the Hampshire Chalk valley.

But Haviland's work bore good fruit, for it opened up a new line of investigation in malignant disease by calling attention to the local incidence of cancer. Speaking only of England, for equally good work has been both in France and Germany, Dr. Law Webb has made the subject his own at Ironbridge, Dr. Lloyd Jones at Cambridge, Dr. Nason at Nuneaton, Mr. Giffard Nash at Bedford, whilst I have had opportunities of investigating villages in the Fens and in Northamptonshire. "Briefly," says Dr. Lloyd Jones in his excellent contribution to the topographical distribution of cancer, "the following are the points upon which, in each case, several of the most trustworthy authors are agreed: (1) that cancer picks out certain districts, and that there are areas of immunity and cancerous areas; (2) that immune areas may lie close beside cancerous areas; (3) that malignant disease is most common along the banks of rivers or streams; (4) that elevated districts or plateaux are more free from cancer while low-lying districts carry more liability to its inroads; (5) that isolated cancer houses or groups of such houses occur in certain districts; (6) that cancer houses often occur near to woods, orchards or forests of firs; (7) that persons living upon flooded and clay districts and retentive soils are especially liable to its ravages; (8) that limestone districts are comparatively free from cancer but do not confer absolute immunity; (9) that cancer may be disseminated by means of water, that it depends upon the presence of decaying animal or vegetable matter or upon sewage; (10) lastly, the suggestion is thrown out by some observers that insects may play a part in the dissemination of malignant disease."

The results here summarised are interesting, and show the amount of thought and work which has been devoted to this side of the cancer question, but they have not yet added anything to our knowledge of the cause of malignant disease. Indeed, my own observations in two widely separated villages where cancer was thought to be unduly prevalent showed that in one village there had been a real increase in cancer, whilst in the other the increase was only apparent. The surroundings of the village in which there had been a real increase in cancer were almost precisely those which had been predicated by Mr. Haviland; but, in the other case, where the surroundings were of a very different nature, the apparent increase seemed to be due to the fact that only the very old and the very young died in the village, because the larger proportion of those who should have been the adult population migrated in search of work.

In the pursuit of these investigations it became apparent that cancer was not only unequally distributed as regards a district, but that in a given district where the cancer mortality was unduly high it was unequally distributed amongst the houses. In some houses successive occupants who were not related to each other died of malignant disease, so that it became possible to speak of "cancer houses." Some of these houses were old, others were comparatively new and their history was often well known locally. But so far as appears at present none of these houses have any features in common, for some are cottages and some are well-built houses of brick or stone. As I have seen them, however, they are usually damp, either from the nature of the soil upon which they are built, or by reason of surrounding trees or water in their immediate neighbourhood. In one case the house is built over a stream which runs down from some high land just behind the house, and in another there is, I am told, the greatest difficulty in preventing the walls and furniture from becoming mouldy.

### The Era of Co-operation.

Until quite recently all investigations upon cancer were carried out by individual workers, each selecting his own line of work without any regard to what was being done by his fellow-workers in the same field. But of late years there has arisen a decided tendency to work in association and under the guidance of some central authority. The co-operative period in this country seems to have begun about 1885 when, at the instigation of Mr. Jonathan Hutchinson, a series of collective investigations upon disease was made by medical practitioners in different parts of the country. Cancer was one of the diseases selected for elucidation by this method, and for the sake of simplicity the inquiry was limited to cancer of the breast. The points which were taken were the influence of inheritance, the influence of residence or locality, and the effect of diet. Mr. Butlin summed up the results which had been obtained in his report published on February 26th, 1887. He says that 210 returns were obtained from 111 observers. The intention of the committee was to apply particularly to men in general practice, and of those who replied

25 resided in the Metropolis and 75 in various parts of the country. Of the three great divisions of inquiry, 184 of the 210 returns contained definite statements. Of these, 116 were to the effect that there was no family history of cancer; 68 that cancer had occurred in other members of the patient's family. The proportion, therefore, is more than one in three, a fact of great interest when it is remembered that in the discussion on cancer at the Pathological Society in 1873, Sir James Paget stated that since his practice had become exclusively or almost exclusively private, one in three was the proportion of cases in which it was possible to discover a history of cancer in the family of his private patients.

The question relating to diet was answered in 194 of the 210 returns. Under the heading of general food it is stated that 123 were moderate feeders, 59 small feeders, and 12 large feeders. Under the heading of meat 99 were moderate, 78 small, and 16 large eaters. On the other hand, there was not a single strict vegetarian. Several of the patients are said to have been great eaters of vegetables and fruit; one was almost a vegetarian; one had taken a diet "chiefly vegetarian of late years"; one ate meat only three times a week; and one ate little or no meat. Under the head of stimulants, 161 are said to have been moderate, nine large drinkers and 21 took none at all. Of the nine large drinkers two took spirits habitually but were not drunkards, and one was an habitual drunkard.

As regards locality, the proximity of a river or stream to the residence of the patient is mentioned in rather more than half the total number of cases. In the returns which came from the country the proximity of a river or stream is noticed in 84, and that of a canal or reservoir in six cases. There were also several patients residing close to the seashore. The London returns furnish 25 instances of patients living near to the river bank of the Thames or of the Lea. In 47 of the country returns the river or stream is said to have been subject to a seasonal overflow. It must be noted that some of the returns which do not describe the dwelling of the patient as being situated on or near a river or stream nevertheless state that the soil was damp; and, on the other hand, that several of the returns which note the proximity of a river or stream state that it was distant from a quarter to half a mile from the patient's residence. But the influence of a river, especially a river of large size, is often felt at a far greater distance from its bank than a quarter or half a mile.

In 1899 a committee was appointed by the Birmingham and Midland Counties Branch of the British Medical Association to investigate the influence of locality on the prevalence of malignant disease in the counties of Warwick, Stafford, Salop, and Worcester. But it was not until the beginning of the present century that public interest was sufficiently awakened to allow of really organised work. The initiative was taken by Germany, where a representative committee was formed by the selection of members from different countries to inquire into the cause of cancer. A similar committee was formed in America under the title of "The Cancer Committee to the Surgical Department of the Harvard Medical School." This committee is working on thoroughly scientific lines under the general direction of Dr. E. H. Nichols. It has already published two annual reports which show that it is engaged in working out on histological and bacteriological lines the truth or otherwise of the parasitic theory of cancer. Dr. Nichols summarises the work of the year 1901 in the following sentences:

It has been claimed by the adherents of the theory of the parasitic origin of cancer that (1) a proliferation of epithelial cells analogous to the lesions seen in cancerous tumours can be produced by certain well-known protozoa (nodules caused by the coccidium oviforme); (2) certain skin lesions characterized by epithelial cell proliferation are due to the action of a so-called protozoon (molluscum contagiosum); (3) blastomycetes are constantly present in human cancers, and are the cause of the lesion; (4) by experimental inoculations of animals with "blastomycetes," true epithelial or cancerous nodules can be produced; (5) finally, the well known endocellular bodies seen in the protoplasm of cancer cells have a definite morphology, are "parasites," and the cause of cancer.

It has been the object of the investigators at the Harvard medical school to study each of these questions, and their latest conclusions were published in February, 1902. As a result of the lines of work pursued by them (under the direction of the Cancer Commission), it is concluded that:—(1) The lesion produced by the coccidium oviforme is essentially a process of chronic inflammation, and is not analogous to the lesion seen in cancer. (2) The lesion in molluscum contagiosum is characterised by certain changes in the epidermis, is not due to the action of a protozoon, and is not analogous to cancer. (3) The so-called "blastomycetes" ("saccharomycetes") of Sanfelice and Plimmer are torulæ. (4) The lesions produced by these "blastomycetes" (torulæ) are essentially nodules of peculiar granulation tissue, they are not cancerous, nor in any sense true "tumours." (5) "Blastomycetes" are not constantly present in human cancers. (6) The peculiar bodies seen in the protoplasm of cancer cells are not parasites, nor the cause of the lesions, but probably are in part at least a typical stage of the process of secretion by glandular epithelium.

The results thus formulated show that the American co-operative investigation is doing good work and is clearing the way for further advances.

In England a Cancer Research Fund was founded in 1902, the first annual meeting being held on July 30th, 1903, Mr. Balfour taking the chair, in the absence of the Prince of Wales, President of the Fund. Dr. Bashford, the general superintendent, read his first year's report. He showed that the work of the Fund is to be conducted upon the widest scientific lines. The term "cancer" for the purposes of research is to be held to include all the malignant new growths, whether occurring in man or the lower animals, and members of the veterinary profession and of the Agricultural Board have been enlisted as workers. During the last year 31 cases of malignant disease have been identified in animals, and a series of epitheliomata from horses, cows, and dogs, and some specimens from the latter animal presenting the histological features of rodent ulcer have also been examined. The subject of electrical treatment of cancer has also come within the purview of the work, and it is very necessary to have an authoritative statement of its value in cases of cancer. All classes have still a feeling of veneration for electrical phenomena, a feeling which is doubtless the survival of many thousand years of nature worship, and they are well contented to undergo a course of electrical treatment in the fullest belief that they will receive benefit. Electro-therapeutics in the hands of the unscrupulous are liable, therefore, to be grossly abused, whilst at the same time by encouraging false hopes and postponing the time for a surgical operation they may cause the untimely death of the patient. Dr. Bashford says that 400 cases have been collected in which electrical treatment in one form or another has been applied to patients suffering from cancer, and the report is favourable to the treatment of rodent ulcer by $x$-rays. With regard to the other varieties of malignant disease, it is said to be very difficult to estimate the effect of electrical treatment, and that at present no results have been brought forward which establish the efficiency of any form of electrical treatment as a curative agent in cases either of sarcoma or of cancer.

### The Problems of Cancer.

Dr. Bashford, however, has done more than summarise the results of past work, for he has recently published a short but important contribution to the subject of cancer research under the title "The Problems of Cancer." He points out that our knowledge of malignant new growths is at the present time almost limited to a recognition of the facts which present themselves for investigation in the pathological laboratories obtaining their material from the post mortem or operating theatres of hospitals in the most civilised parts of the world. But many other lines of inquiry are open to the student of malignant disease, especially biological and biochemical. The conditions of growth of cancerous tissue as distinct from the problems of the genesis of malignant new growths have been hardly touched upon, except in a purely speculative fashion, and in the latter direction to a degree out of all proportion to the meagreness of the facts accumulated. Sir James Paget and Mr. Roger Williams were once much interested in the pathology of gall-nuts and of vegetable tumours in relation to bud formation. But of late years very little has been done in connection with vegetable pathology which might shed a light on the question of malignant disease in animals. The question of cancer has been looked upon somewhat too exclusively as a problem of purely human pathology, and this is due in part to the fact that veterinary surgeons have until quite recently maintained that cancer in animals was of rare occurrence ; a statement made no doubt in all good faith, but the result partly of ignorance and partly of the fact that few animals are allowed to attain to the limit of age at which cancer is most likely to occur. It is now well established that the domestic animals are by no means free from malignant disease in its various forms, but as yet we are as ignorant of its occurrence in wild animals as we are in wild men. Indeed, an examination of malignant disease in animals may prove to be of the greatest value, for the length of the human life-cycle has been a great impediment to the solution of many problems which can be easily investigated in short-lived animals. Old age in the domestic animals is attained at 14 to 20 years or less, and Dr. Bashford says "it is obvious, therefore, that identical manifestations first presenting themselves in any number in mankind after 40 years of age, and in the common domestic animals at 14 to 20 years, must pass through the latent non-manifest stages in a much shorter period in the latter. It may be that this latent period will be found to be in strict proportion to the duration of life, and in this case it will be different and definite for each species in the same way as is the period of gestation. The fact that such pronounced variations occur in the periods within which identical processes manifest themselves in man and in different species of animals would indicate the important dependence of the development of malignant new growths on circumstances innate in each species of organism, and on the biological laws peculiar to, and controlling the growth of, each species of animal. As has been shown, this is already borne out by the work of Jensen, who has been able to graft the epitheliomatous growths of mice upon mice with successful results in more than 100 instances, and in 19 generations of animals. Dr. Bashford thinks further that a comparative study of the age-incidence of cancer among domestic and wild animals of varied species will certainly give comprehensive information on the incidence of cancer to the rapidity with which maturity is attained. Light may also be thrown on the reasons underlying the higher cancer-rate of later life. For the same reason exact observations on the age-incidence in the males and females of differing races of mankind, who attain maturity at varying ages is desirable. A comparison of the age-incidence of cancer in men as contrasted with that in women who reach maturity more rapidly, might also be of value—for example in India, where puberty is attained at an early age, and the child-bearing period is said to be over at or about the thirtieth year.

A comparison of the ideas suggested for collective investigation in 1885 and in 1903 will show the advance which has been made in the interval in regard to malignant disease. In 1885 cancer was looked upon as a disease which only concerned medical men : in 1903 it is recognised as a subject which extends far beyond the province of medicine, for it trenches upon general biology, ethnology, sociology, zoology and embryology. No single individual is competent to deal with all these branches of knowledge, just as there is no one now living who is competent to lecture upon chemistry, botany, and medicine, as did many of the older professors in our universities and medical schools. It is very necessary, therefore, that a central board should be established where advice may be given and information may be obtained as to the progress of investigation in different directions by those who are well acquainted with the work which is being done in the different fields. Such an organisation, however, requires money, and although a good beginning has been made the honorary treasurer is already complaining that the pecuniary support is still insufficient to place the undertaking on a firm financial basis. There is little doubt that as the work proceeds and the utility of the fund becomes apparent to the general public, sufficient money will be forthcoming to make it a permanent body, and the generous donation of £25,000 by Mr. W. W. Astor has already done much to relieve the anxiety expressed by the Treasurer of the Cancer Research Fund.

Prospects of Cure.

The only treatment at the present time which holds out any prospect of cure in cancer is complete removal of the growing mass with the adjacent lymphatic glands, and to be of any real service this operation must be performed at the earliest possible moment after the diagnosis of malignant disease has been made. A complete and thorough removal often means freedom from recurrence, either permanently or for periods which are measured rather by years than by months. Yet we are slowly accumulating evidence to show that other methods of treatment will be as available and may be even more effective in the near future. It has been known for a long time that undoubted cancers sometimes dwindle instead of growing. The removal of the ovaries in women has undoubtedly in some cases an influence in arresting the growth of cancer of the breast. The injection of an appropriate serum, there is reason to believe, has actually cured cases of fungating cancer which were thought to be beyond the reach of surgical interference. In each case the rationale of the cure has been the same—cessation of growth in the epithelial cells with increased formation of fibrous tissue. These are the lines of treatment which must be followed in the future, and which will probably lead us to an explanation of the cause of cancer, and may at the same time afford a rational cure. But at present all treatment except that by direct removal is in an experimental stage; the other methods are only tried in desperate cases, their results at present are too uncertain to allow of the formulation of rules, and their use is consequently purely empirical.

